# Supplementary material for: Free spermidine evokes superoxide radicals that manifest toxicity
Source: eLife. 2022 Apr 13;11:e77704. doi: 10.7554/eLife.77704 (PMC9038194; doi:10.7554/eLife.77704)

HPLC profiles of dansylated samples

(**Peak 1**: Hexane diamine int STD; **Peak 2**: Spermidine)

WT samples (triplicate)

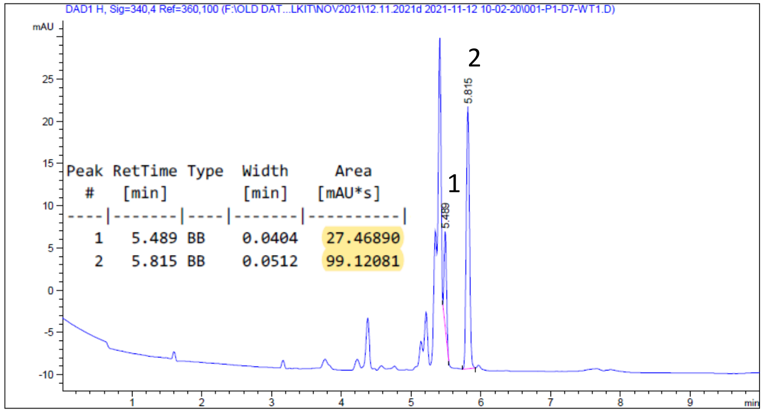

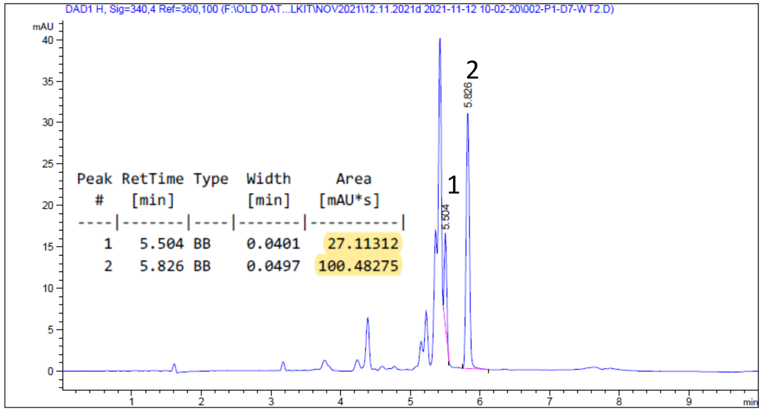

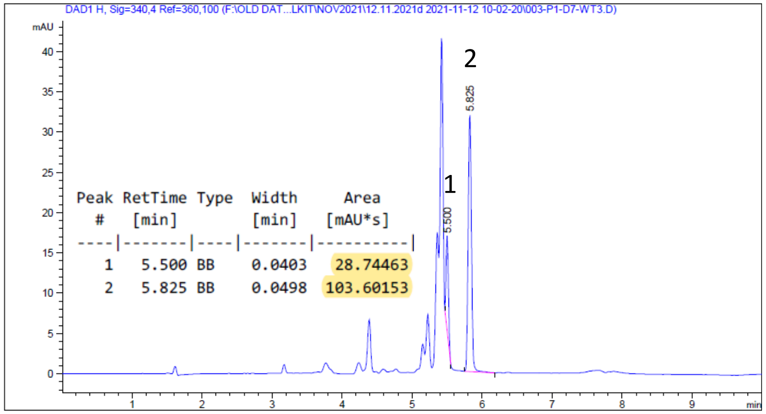

WT+spermidine samples (triplicate)

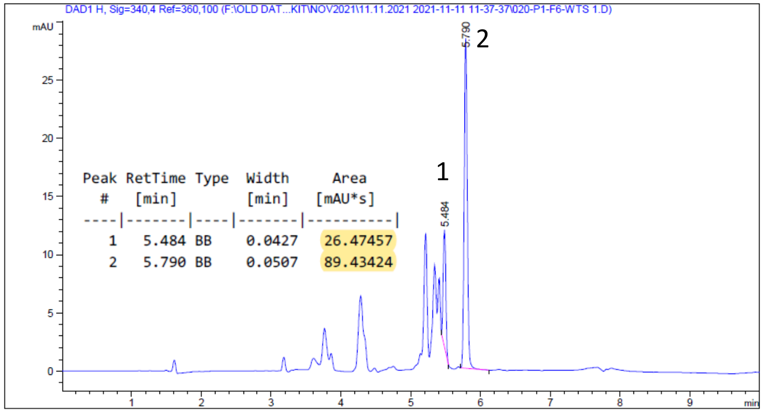

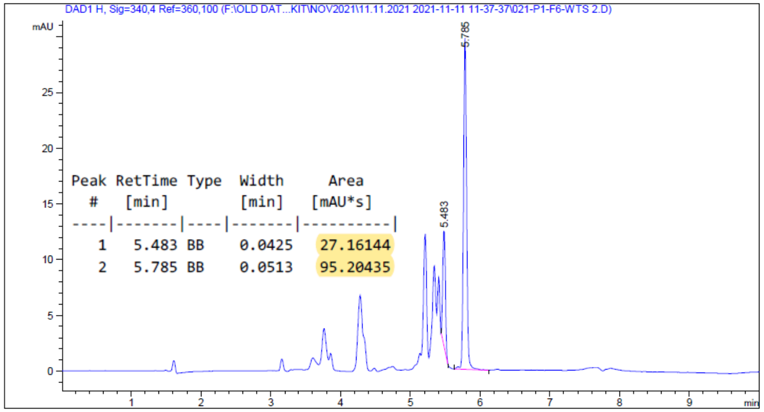

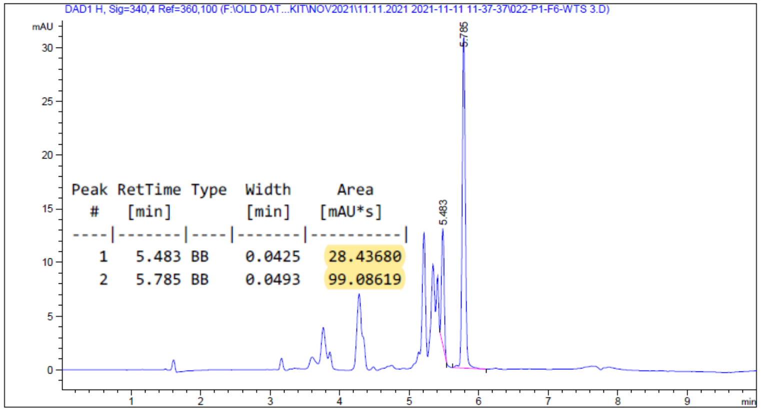

HPLC profiles of dansylated samples

(**Peak 1**: Hexane diamine int STD; **Peak 2**: Spermidine)

Δ*speG* samples (triplicate)

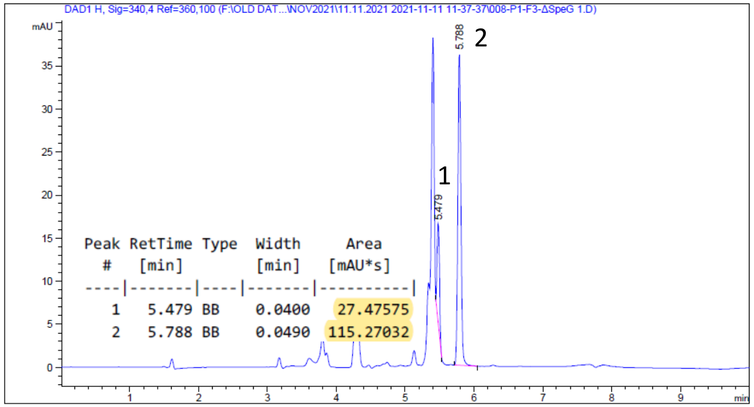

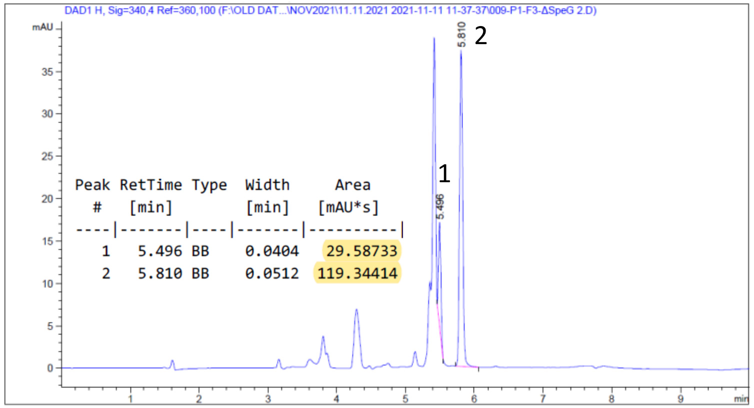

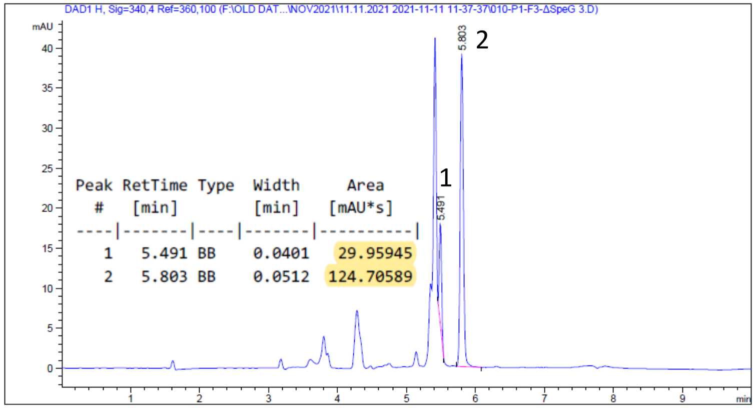

Δ*speG* +spermidine samples (triplicate)

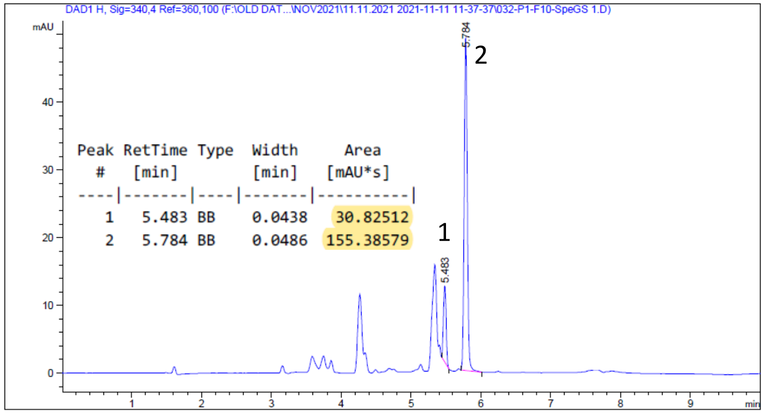

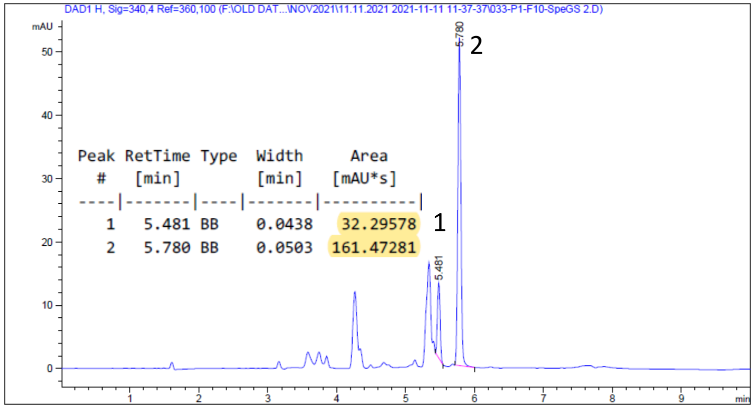

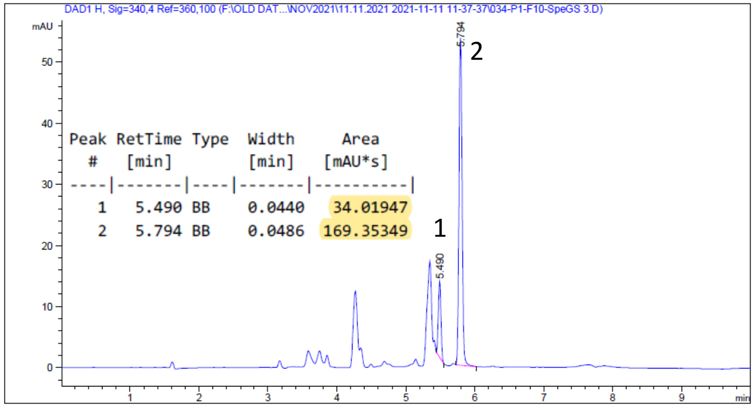

HPLC profiles of dansylated samples

Δ*speE* samples (triplicate)

(**Peak 1**: Hexane diamine int STD; **Peak 2**: Spermidine)

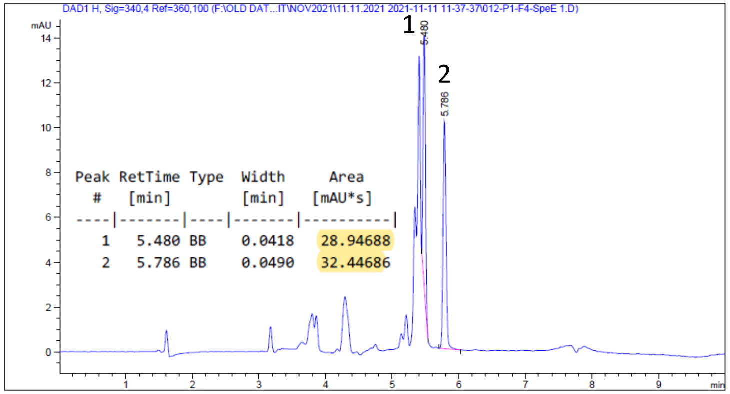

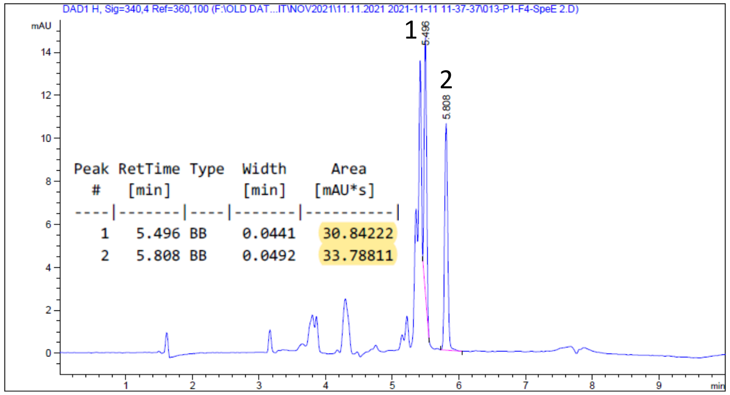

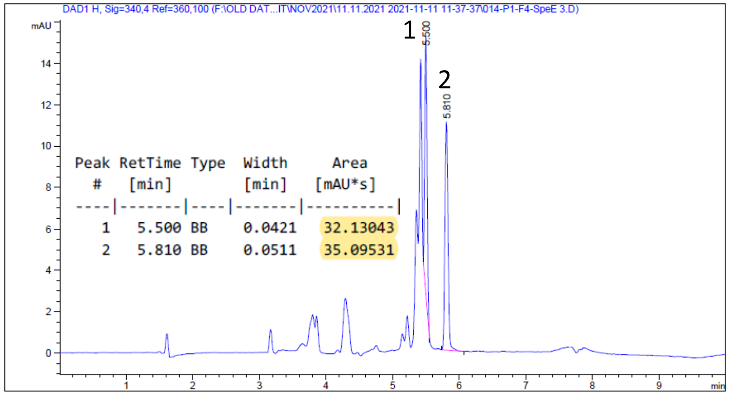

Pure dansylated-spermidine peak

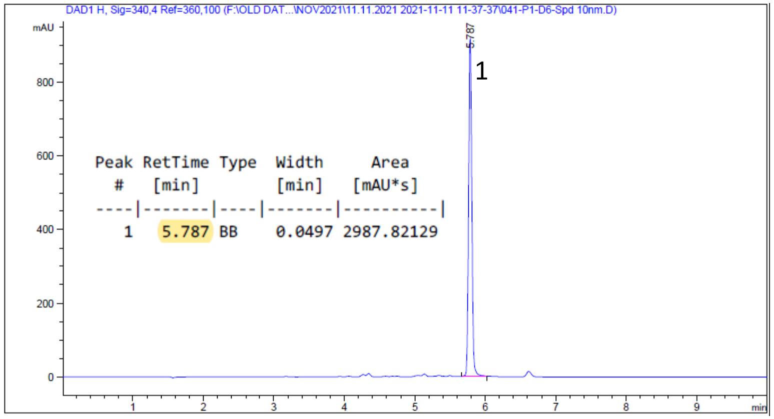

Pure dansylated-Hexane-diamine peak

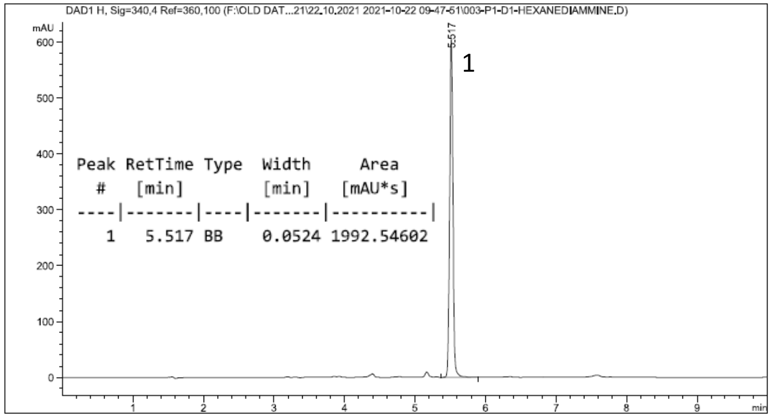

Supplement: Figure 1—figure supplement 1—source data 2. [file elife-77704-fig1-figsupp1-data2.pdf]
