# Supplementary figures and images for: Free spermidine evokes superoxide radicals that manifest toxicity
Source: eLife. 2022 Apr 13;11:e77704. doi: 10.7554/eLife.77704 (PMC9038194; doi:10.7554/eLife.77704)

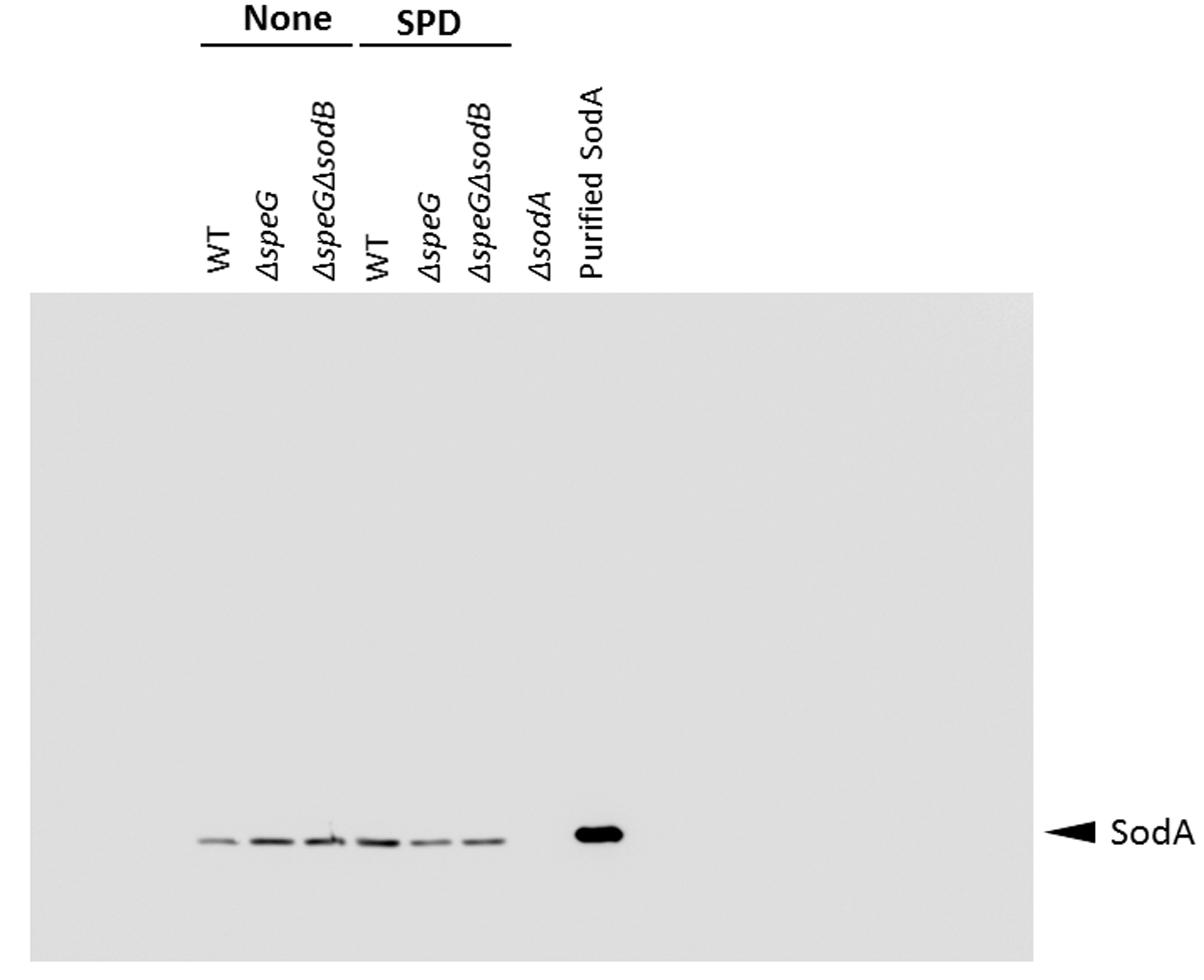

Supplement: Figure 4—source data 3. [file elife-77704-fig4-data3.zip › Figure 4-Source data 3.TIF]

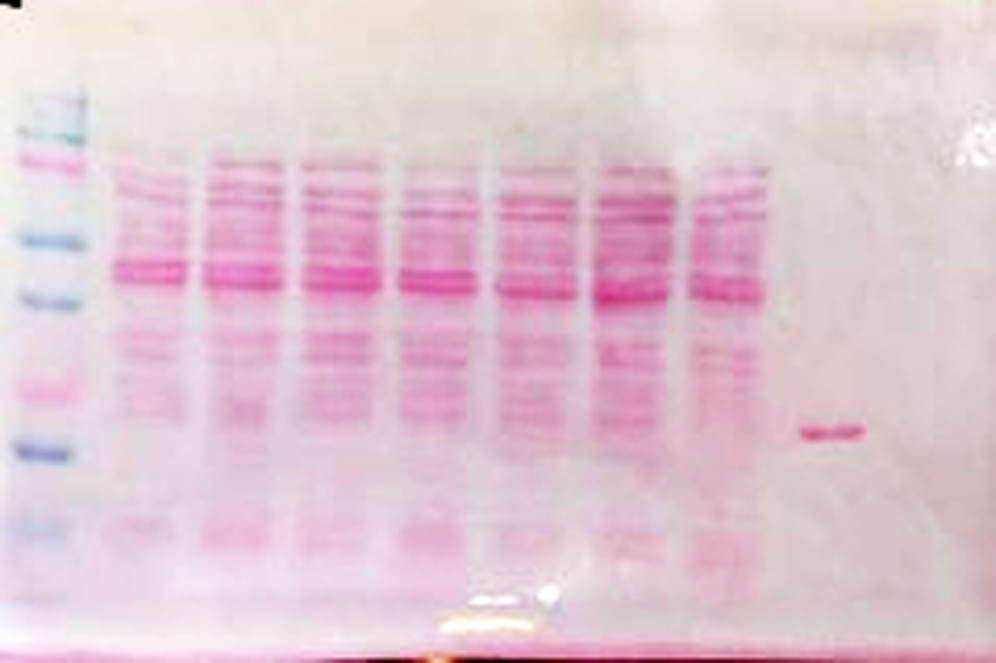

Supplement: Figure 4—source data 4. [file elife-77704-fig4-data4.zip › Figure 4-Source data 4.tif]

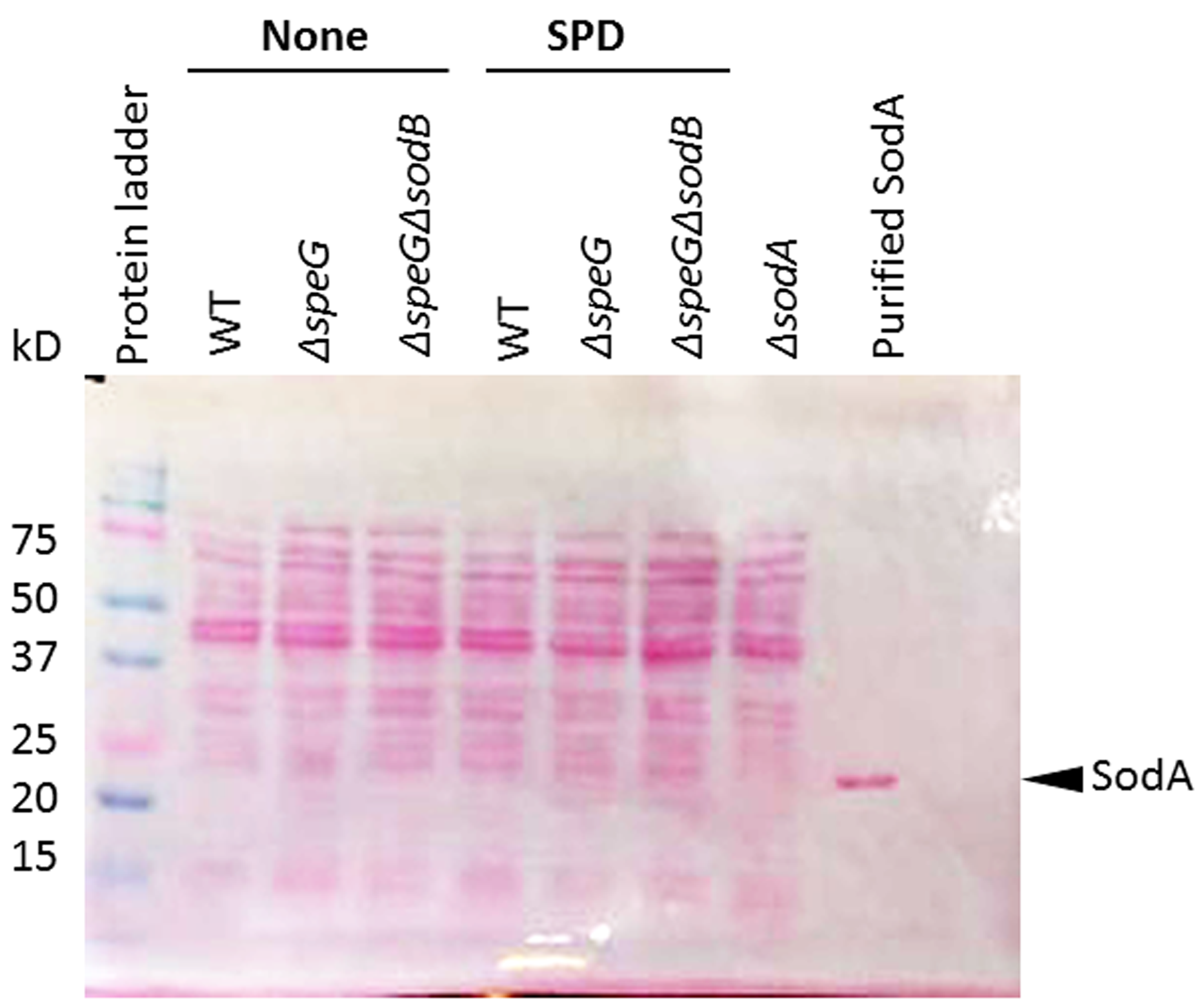

Supplement: Figure 4—source data 5. [file elife-77704-fig4-data5.zip › Figure 4-Source data 5.TIF]

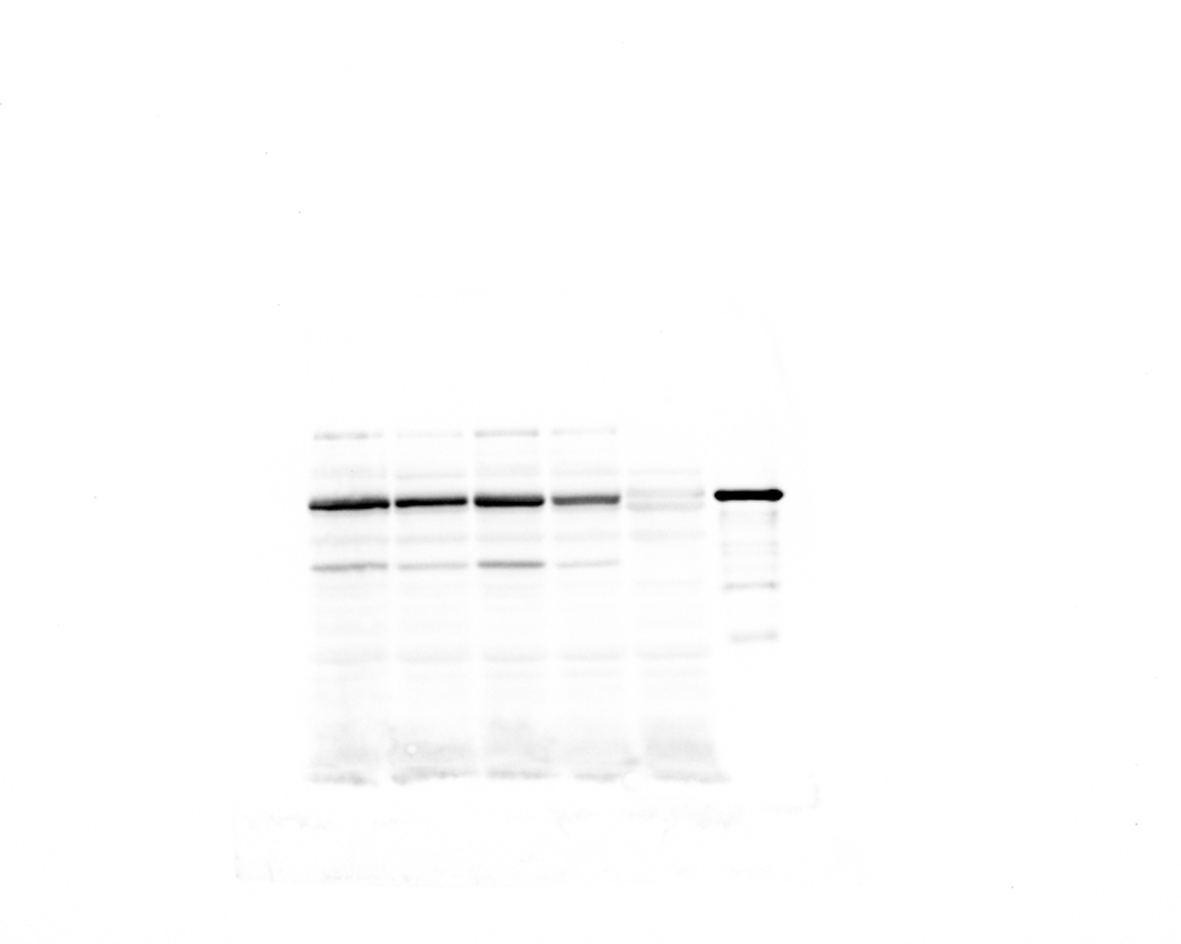

Supplement: Figure 4—source data 6. [file elife-77704-fig4-data6.zip › Figure 4-Source data 6.tif]

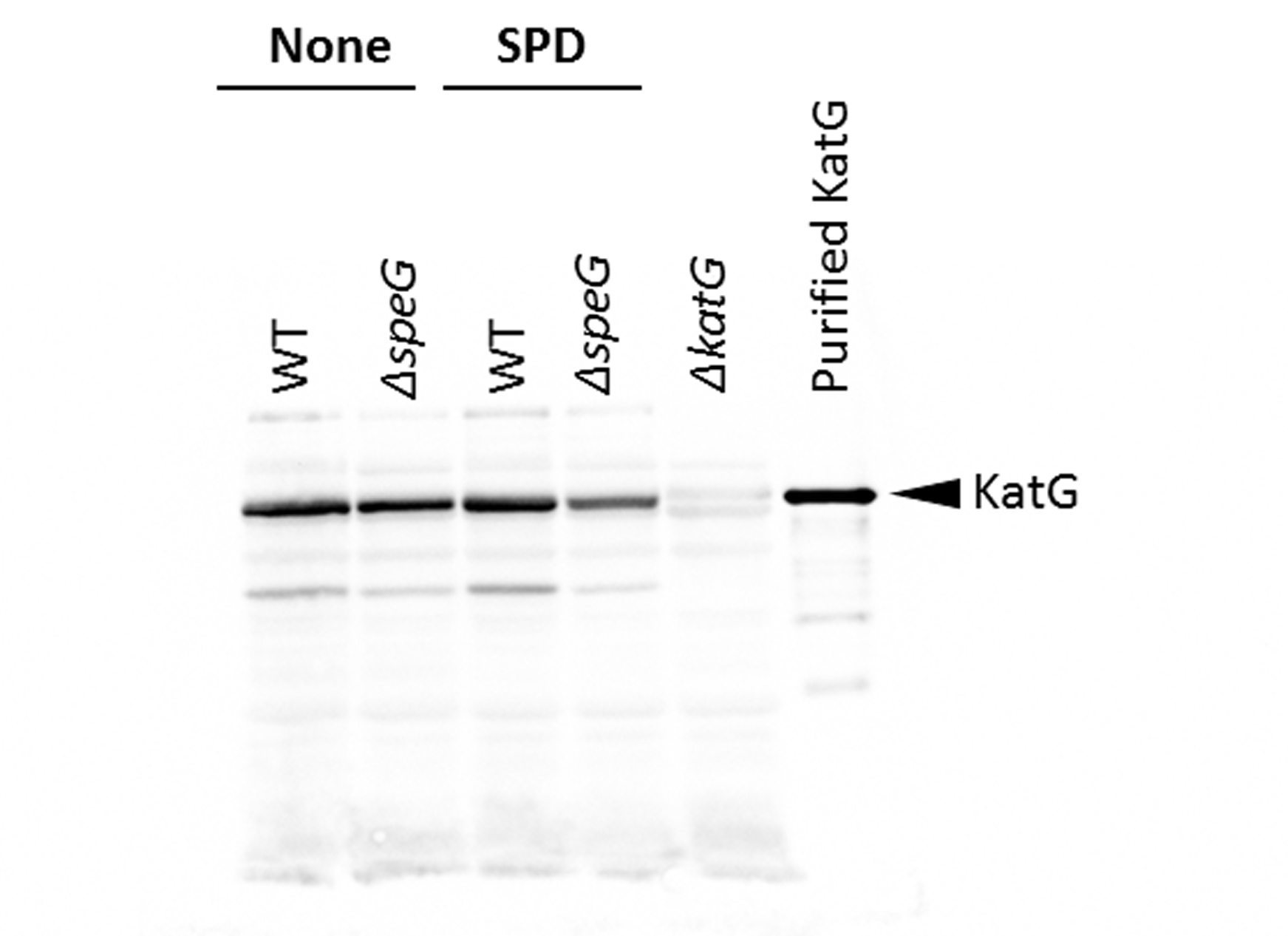

Supplement: Figure 4—source data 7. [file elife-77704-fig4-data7.zip › Figure 4-Source data 7.TIF]

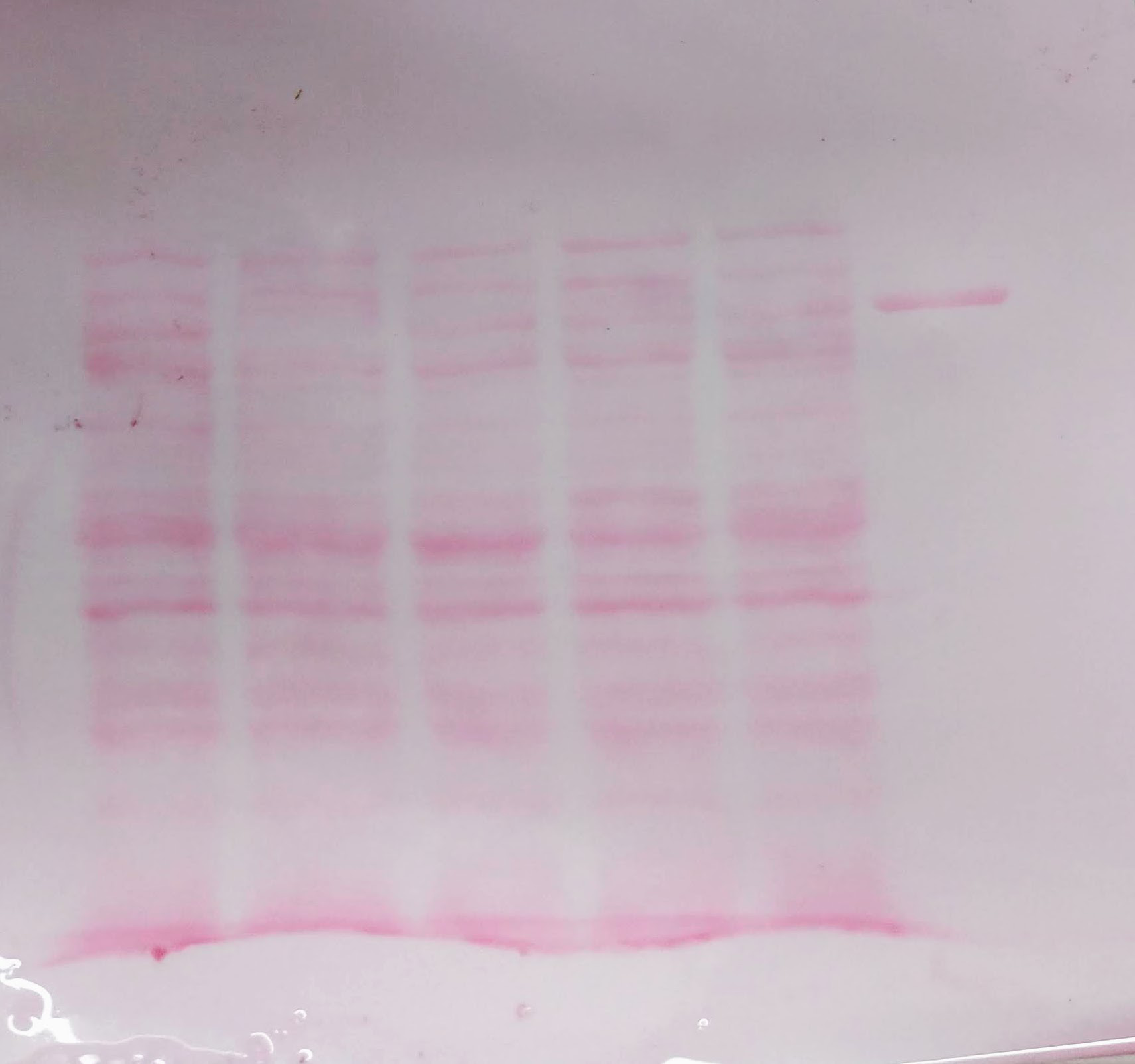

Supplement: Figure 4—source data 8. [file elife-77704-fig4-data8.zip › Figure 4-Source data 8.tif]

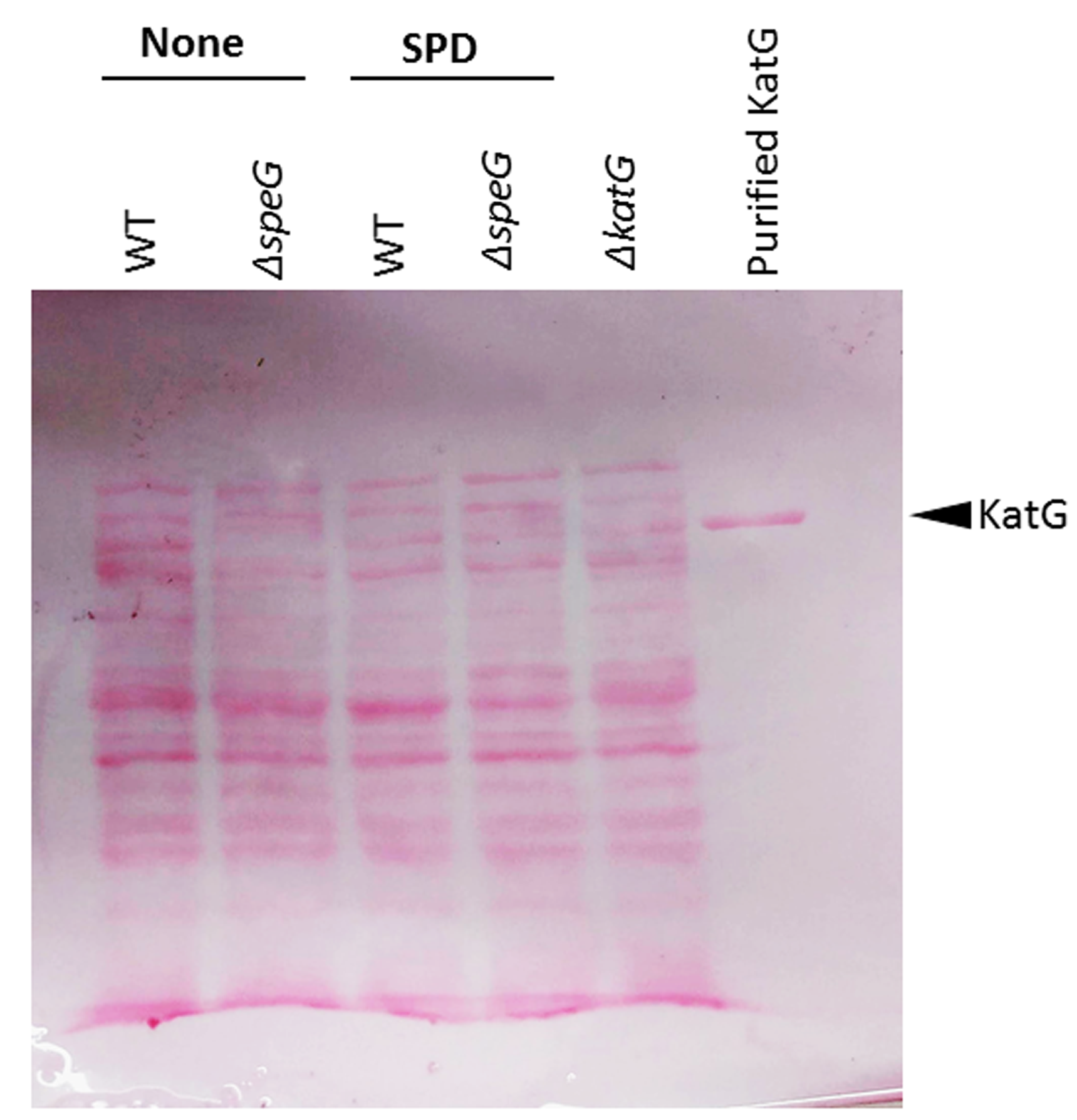

Supplement: Figure 4—source data 9. [file elife-77704-fig4-data9.zip › Figure 4-Source data 9.TIF]

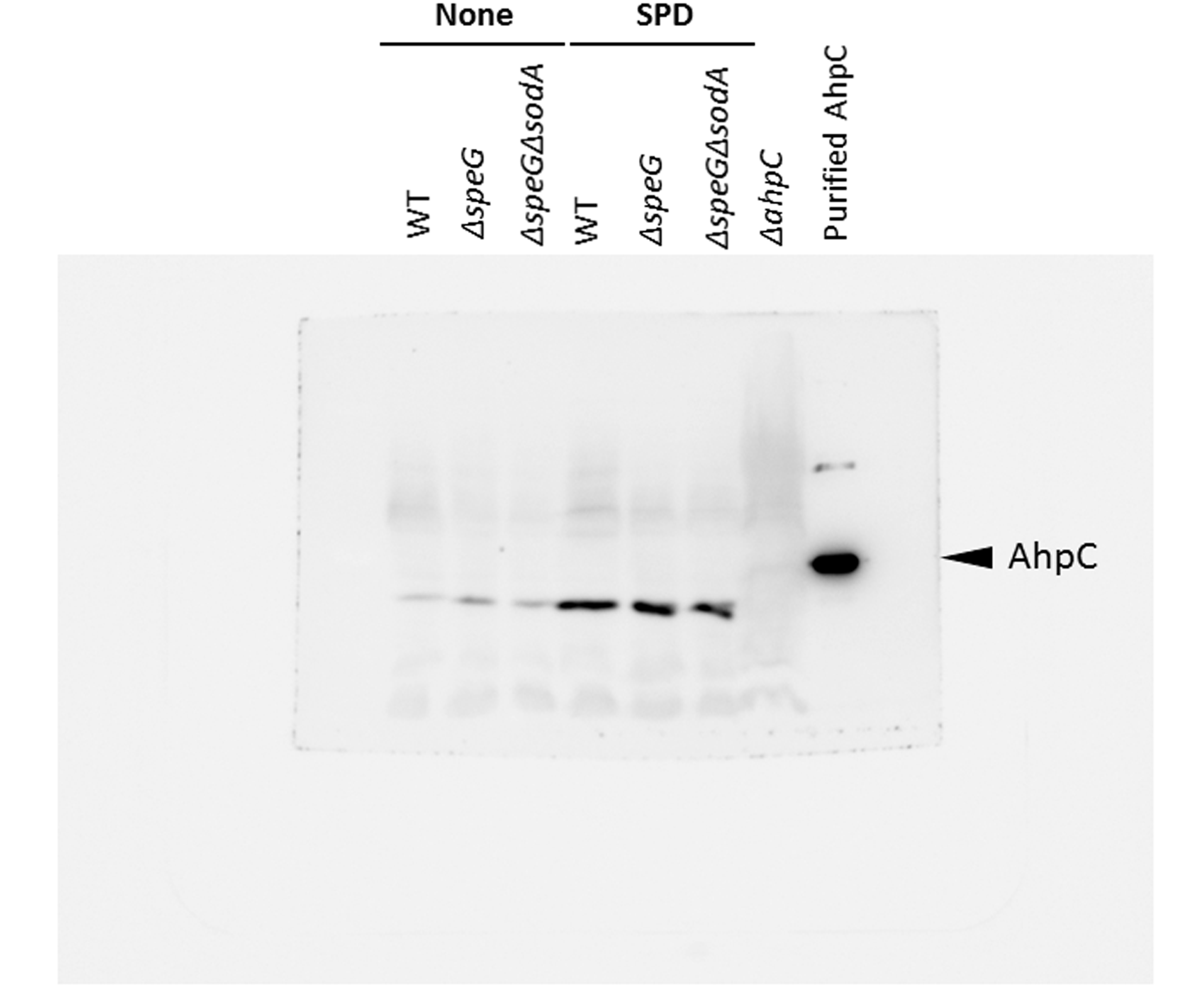

Supplement: Figure 4—source data 11. [file elife-77704-fig4-data11.zip › Figure 4-Source data 11.TIF]

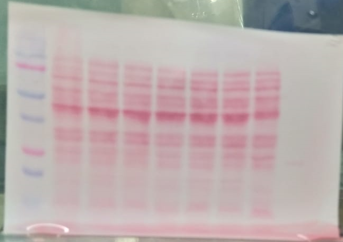

Supplement: Figure 4—source data 12. [file elife-77704-fig4-data12.zip › Figure 4-Source data 12.tif]

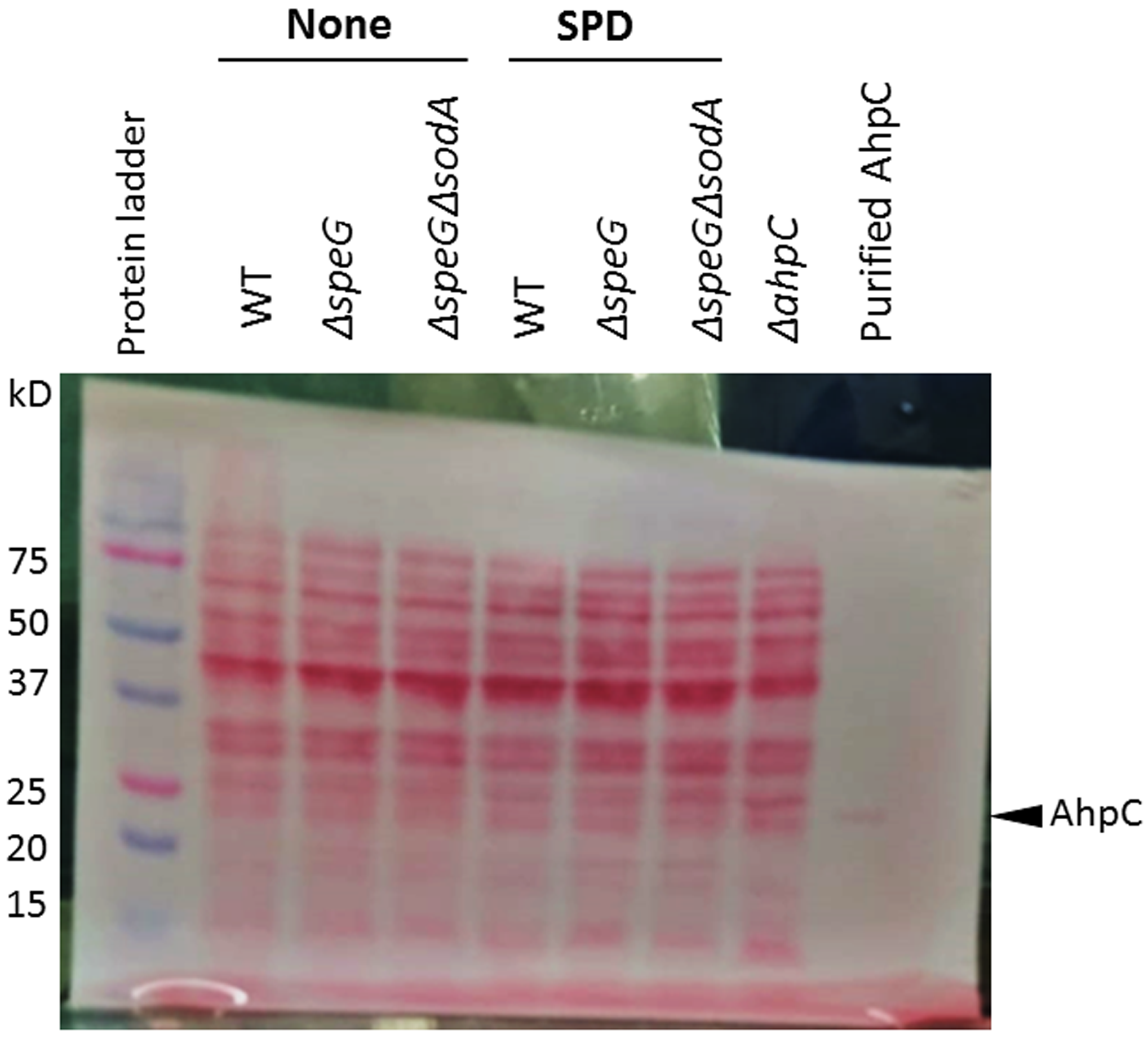

Supplement: Figure 4—source data 13. [file elife-77704-fig4-data13.zip › Figure 4-Source data 13.TIF]
