# Supplementary material for: Free spermidine evokes superoxide radicals that manifest toxicity
Source: eLife. 2022 Apr 13;11:e77704. doi: 10.7554/eLife.77704 (PMC9038194; doi:10.7554/eLife.77704)
Supplement: Supplementary file 3. [file elife-77704-supp3.docx]

**Supplementary file 3. Oligonucleotide primers used in this study.**

| **Cloning Primers** | **Nucleotide sequence** | **Description** |
| --- | --- | --- |
| DG12 | GCTACACATATGAGCTATACCCTGCC | Forward primer with NdeI site |
| DG13 | GCACCGAAGCTTATTTTTTCGCCGC | Reverse primer with HindIII site |
| DG14 | CAATCAGCTAGCATGTCATTCGAATTACC | Forward primer with NheI |
| DG16 | CGATCAAGCTTATGCAGCGAGATTTTTC | Reverse primer with HindIII |
| RM7 | GCAGTACATATGAGCACGTCAGACG | Forward primer with NdeI |
| RM8 | GCTTACAAGCTTTTACAGCAGGTCGAAACG | Reverse primer with HindIII |
| DG9 | GTCACGATCATATGTCCTTGATTAACACC | Forward primer with NdeI |
| DG10 | CTGATGAGGATCCTTAGATTTTACCAACCAG | Reverse primer with BamHI |
| RK3 | CGAGTGCCATATGCCAAGCGCCCACAGTGT | Forward primer with NdeI |
| RK4 | CGAGTGGAATTCCTATTGTGCGGTCGGCTTC | Reverse primer with EcoRI |
| RK55 | GAGTGCCATATGGCGGTAACGCAAACAGCC | Forward primer with NdeI site |
| RK56 | CGAGTGGAATTCTTACTCAAACTCATTCCAG | Reverse primer with EcoRI site |
| **RT Primers** | **Nucleotide sequence** | **Description** |
| sodAF | GCTAACCACAGCCTGTTCTG | Forward and reverse primer pairs for *sodA* gene |
| sodAR | CGCCAGTTTATCGCCTTTCA |  |
| *soxS*F | CGATTACATTCGCCAACGC | Forward and reverse primer pairs for *soxS* gene |
| soxSR | GATCAAACTGCCGACGGAAA |  |
| zwfF | TGTGCCATTCTACCTGCGTA | Forward and reverse primer pairs for *zwf* gene |
| zwfR | CCTTCATCAGGTTGCAGACG |  |
| furF | GGTATCGTCACCCGCCACAA | Forward and reverse primer pairs for *fur* gene |
| furR | TGCGGCAATTTCACGCTGAC |  |
| tigF | TTCGGCGTTGAAGATGGTTC | Forward and reverse primer pairs for *tig* gene |
| tigR | GATCGCCTGAGACTTAACGC |  |
| hscAF | GCTGCCATCTGTTGTTCACT | Forward and reverse primer pairs for *hscA* gene |
| hscAR | TGATAAGGCAGATGCGGATA |  |
| cspFF | AAAGCGGCAAGGGTCTTAT | Forward and reverse primer pairs for *cspA* gene |
| cspFR | TAAACATTGGCAGCTGAAGG |  |
| nsrRF | GGCGGATGACCAGTATTTCT | Forward and reverse primer pairs for *nsrR* gene |
| nsrRR | ACCAATACGTATCGCACTCG |  |
| recAF | GGGCCAGATTGAGAAACAAT | Forward and reverse primer pairs for *recA* gene |
| recAR | GGTCCGTAGATTTCGACGAT |  |
| rplWF | TGAAGAACGTCTGCTGAAGG | Forward and reverse primer pairs for *rplW* gene |
| rplWR | CAGGGTGTTAACGACTTCGAC |  |
| rpsAF | GAAGTTGACGTTGCTCTGGA | Forward and reverse primer pairs for *rpsA* gene |
| rpsAR | CCCTTAACTTTGCCGTTGAT |  |
| iscRF | GCTGATATTTCCGAACGTCA | Forward and reverse primer pairs for *iscR* gene |
| iscRR | GCTAATTACTTCGCCAACGG |  |
| nhaAF | GGGTTGGTTCACTCGAAATC | Forward and reverse primer pairs for *nhaA* gene |
| nhaAR | GCACAATCATCCCACCAATA |  |
| cmrF | CTGAAAGAACTCGGTCGTGA | Forward and reverse primer pairs for *cmr* gene |
| cmrR | TTGCAGCAAGCCATATTCAT |  |
| hdeAF | CCTGTGAAGATTTCCTGGCT | Forward and reverse primer pairs for *hdeA* gene |
| hdeAR | TCCCATTCGCCTTTAACTTT |  |
| dnaKF | ACTCGTATGCCAATGGTTCA | Forward and reverse primer pairs for *dnaK* gene |
| dnaKR | GGTAACGTCCAGCAGCAGTA |  |
| betBF | AACTTCTTCAGCTCCGGTCA | Forward and reverse primer pairs for *betB* gene |
| betBR | GCCGAAGTTAGTTTGCGGAT |  |
